# Provision of nitrogen as ammonium rather than nitrate increases silicon uptake in sugarcane

**DOI:** 10.1093/aobpla/plu080

**Published:** 2014-12-01

**Authors:** Malcolm G. Keeping, R. Stuart Rutherford, C. Sewpersad, Neil Miles

**Affiliations:** 1 South African Sugarcane Research Institute, Private Bag X02, Mount Edgecombe 4300, South Africa; 2 School of Animal, Plant and Environmental Sciences, University of the Witwatersrand, Johannesburg 2050, South Africa; 3 School of Life Sciences, University of KwaZulu-Natal, Durban 4000, South Africa; 4 School of Agricultural, Earth and Environmental Sciences, University of KwaZulu-Natal, Pietermaritzburg 3201, South Africa

**Keywords:** Ammonium, calcium silicate, nitrate, nitrogen, pH, rhizosphere, silicon uptake, sugarcane.

## Abstract

Silicon (Si) is important in mitigating abiotic and biotic plant stresses, yet many agricultural soils, such as those of the rainfed production areas of the South African sugar industry, are deficient in plant-available Si, making Si supplementation necessary. However, Si uptake by sugarcane (*Saccharum* spp. hybrids) is limited even where silicate amendments improve soil Si status. Rhizosphere pH, which can affect Si uptake, can be manipulated using different N-form fertilizers. We tested whether (i) fertilization with $\text{N}{{\text{H}}_{4}}^{+}$ (rhizosphere acidification) increased Si uptake compared with $\text{N}{{\text{O}}_{3}}^{-}$ (rhizosphere alkalinization); and (ii) uptake differed between an N-efficient, more acid-tolerant cultivar (N12) and an N-inefficient, less acid-tolerant cultivar (N14). Two pot trials with low-Si soil were fertilized with calcium silicate (Ca_2_SiO_4_) slag, plus N from ammonium sulphate [(NH_4_)_2_SO_4_], ammonium thiosulphate [(NH_4_)_2_S_2_O_3_] and calcium nitrate [Ca(NO_3_)_2_] (Trial 1) or N from (NH_4_)_2_S_2_O_3_ and Ca(NO_3_)_2_ only (Trial 2). Trial 2 included cultivars N12 and N14. Nitrate treatments significantly increased soil pH and soil Si compared with $\text{N}{{\text{H}}_{4}}^{+}.$ However, $\text{N}{{\text{H}}_{4}}^{+}$ treatments significantly increased leaf and stalk Si content compared with $\text{N}{{\text{O}}_{3}}^{-},$ reflected in a significant negative relationship between soil pH and leaf Si. Acid-extracted soil Si was negatively related to leaf and stalk Si, likely due to adsorption of silicic acid to soil surfaces under higher pH of the $\text{N}{{\text{O}}_{3}}^{-}$ treatment and its reduced availability for plant uptake. We conclude that $\text{N}{{\text{H}}_{4}}^{+}$ increased Si uptake into leaf and stalk, and propose that reduced rhizosphere pH solubilized Si from Ca_2_SiO_4_ and increased silicic acid availability for plant uptake. By contrast, $\text{N}{{\text{O}}_{3}}^{-}$ may have reduced Si uptake due to adsorption of Si to soil surfaces at higher pH. Our results indicate that ammoniacal fertilizers, such as (NH_4_)_2_SO_4_ and urea, have potential for promoting dissolution of applied Ca_2_SiO_4_ and subsequent uptake of Si by sugarcane.

## Abstract (precis)

Silicon's role in ameliorating a range of biotic and abiotic plant stresses is beyond doubt, yet means to maximise its uptake via the roots from applied silicon sources and thereby enhance crop yields have not been fully explored. Our study found that reduction of rhizosphere pH through provision of nitrogen fertilizer to sugarcane as ammonium rather than nitrate increased silicon uptake from a low-silicon soil amended with calcium silicate slag. We propose that ammoniacal fertilizers have potential for enhancing the solubilisation of silicate slags by acidifying the rhizosphere and increasing silicic acid solubility and availability for plant uptake.

## Introduction

Silicon (Si) is the second most abundant element in the Earth's crust after oxygen, where it may constitute 28 % of the soil mass as silicate minerals and water-soluble monosilicic acid (H_4_SiOH_4_) (Epstein 2001). However, in the humid tropics and subtropics high rainfall and temperatures have subjected soils to intensive chemical weathering over the millennia, leading to a loss of soluble Si (desilication) through leaching or erosion, and the development of soils with low base saturation and that are high in aluminium and iron sesquioxides (McKeague and Cline 1963*c*; Savant *et al.* 1997*a*; Epstein 2001). Furthermore, crops such as rice (*Oryza sativa*) and sugarcane (*Saccharum* spp. hybrids), which are Si accumulators (>1.0 % shoot Si) (Ma *et al.* 2001; Guntzer *et al.* 2012), are capable of removing up to 470 and 500 kg Si ha^−1^ annum^−1^, respectively, on productive, Si-rich soils (Ross *et al.* 1974; Savant *et al.* 1997*b*, 1999). On weathered low-Si soils, intensive farming of such crops can therefore aggravate the depletion of plant-available reserves of Si (i.e. silicic acid) and result in crops deficient in this nutrient (Savant *et al.* 1997*a*; Berthelsen *et al.* 2001*b*; Guntzer *et al.* 2012).

Increasing evidence of the role of Si in plant adaptations that mitigate a range of abiotic and biotic stressors (for recent reviews, see Liang *et al.* 2007; Epstein 2009; Romero *et al.* 2011; Marafon and Endres 2013) has highlighted the need to provide certain crops with supplemental Si, to moderate yield losses where plant-available soil Si is low (<10 mg L^−1^; 0.01 M CaCl_2_ extraction) (Berthelsen *et al.* 2001*a*).This is particularly important where there is likely to be some form of plant stress (Winslow *et al.* 1997; Berthelsen *et al.* 2001*b*; Ma *et al.* 2001). In sub-Saharan Africa, including Madagascar, widespread yield reductions and disease susceptibility in rice have been ascribed to Si deficiencies, particularly in acidic, weathered Oxisol and Ultisol soils of highland and humid agro-ecological zones (Winslow 1992; Tsujimoto *et al.* 2014). In South Africa, comparable soil and environmental conditions are associated with leaf Si deficiencies in sugarcane in the rainfed (non-irrigated) areas of production (Meyer *et al.* 1998; Meyer and Keeping 2001; Van der Laan and Miles 2010; Miles *et al.* 2014). Both pot and field experiments have shown that the correction of such deficiencies through Si provision in the form of calcium silicate, blast furnace slags and cement, could significantly increase leaf Si content up to 16 g kg^−1^ dry matter and cane yield up to 53 % above unamended controls and lime treatments applied at the same rate (Du Preez 1970; Meyer and Keeping 2001). The silicate treatments decreased exchangeable Al and Mn, and increased pH to an extent similar to that of lime; hence the increased yields in the silicate treatments were due not only to elimination of toxic amounts of Al and Mn, but also to provision of sufficient Si to optimize yields (Du Preez 1970; Meyer and Keeping 2001). Similar results with sugarcane have been obtained elsewhere in the world in desilicated soils (for reviews, see: Savant *et al.* 1999; Alvarez and Datnoff 2001; Berthelsen *et al.* 2001*b*).

Average leaf Si content of sugarcane grown in coastal and inland non-irrigated areas of the South African sugar industry has, since first recorded in 2001, seldom exceeded the industry threshold (Miles and Rhodes 2013) of 7.5 g kg^−1^ dry matter; in contrast, that of cane in the northern irrigated areas, where soils are less acid (pH > 6.5), ranged from 10 to 25 g kg^−1^ (Van der Laan and Miles 2010). Furthermore, and notwithstanding the results of the experiments described by Du Preez (1970) and Meyer and Keeping (2001), more recent field trials with Si materials applied to low-Si soils (<10 mg L^−1^ Si; 0.01 M CaCl_2_) in the rainfed regions have failed to produce leaf Si values above the 7.5 g kg^−1^ threshold, despite the greatly improved Si status of the soil and the strong liming capacity of the materials used (Keeping *et al.* 2013; Rhodes *et al.* 2013). Keeping *et al.* (2013) listed a number of possible reasons for this, in particular reduced Si solubility at higher pH due to increased adsorption of silicic acid to iron and aluminium sesquioxides (Beckwith and Reeve 1963; Jones and Handreck 1963; McKeague and Cline 1963*b*; Tavakkoli *et al.* 2011). On the other hand, Korndörfer *et al.* (2005) have argued that increases in pH in weathered soil promote the release of colloid-adsorbed Si to the soil solution and the transformation of polysilicic acid into monosilicic acid. In support of this, Oliveira *et al.* (2007) found that an increase in rhizosphere pH through fertilization with nitrogen (N) in the nitrate $(N{{\text{O}}_{3}}^{-})$ rather than ammonium $(N{{\text{H}}_{4}}^{+})$ form increased soil Si solubility and uptake in rice. However, in a 5-year field rotational cropping experiment including sugarcane, corn (*Zea mays*) and kikuyu grass (*Pennesetum clandestinum*), Khalid *et al.* (1978) found that relative amounts of applied Si recovered by plants decreased with increasing soil pH, while that extracted from the soil increased with increasing pH. The influence of soil pH on Si uptake is therefore contentious and further investigation is clearly necessary.

As part of a continuing effort to maximize Si uptake from applied calcium silicate (Ca_2_SiO_4_) sources under the rainfed growing conditions of sugarcane in South Africa, we reasoned that, based on these earlier findings, manipulation of fertilizer N-form may assist in solubilizing Si from applied Ca_2_SiO_4_. Although Ca_2_SiO_4_ is barely soluble in water at neutral pH, it will dissociate in a more acidic (soil) solution (Ma and Takahashi 2002). Hence, we predicted that reduced rhizosphere pH, resulting either from bulk acidification of soil due to nitrification of $\text{N}{{\text{H}}_{4}}^{+}$ supplied as ammonium sulphate [(NH_4_)_2_SO_4_] (Reaction A below) or from H^+^ ion extrusion from the root to balance charges following $\text{N}{{\text{H}}_{4}}^{+}$ uptake (Reaction B below) (Thomson *et al.* 1993; Marschner 1995; Hinsinger *et al.* 2003), was the most likely mechanism whereby Si could be solubilized from Ca_2_SiO_4_, with both processes making silicic acid available in the immediate root environment.

Reaction A (bulk soil acidification):

(1) $$\text{N}{{\text{H}}_{4}}^{+}+2{{\text{O}}_{2}}^{-}\to 2{\text{H}}^{+}+\text{N}{{\text{O}}_{3}}^{-}+{\text{H}}_{2}\text{O}$$

(2) $$2{\text{H}}^{+}+\text{C}{\text{a}}_{2}\text{Si}{\text{O}}_{4}\to {\text{H}}_{4}\text{Si}{\text{O}}_{4}+2\text{C}{\text{a}}^{2+}$$

Reaction B (H^+^ extrusion from root):

(3) $$(\text{N}{\text{H}}_{4}{)}_{2}\text{S}{\text{O}}_{4}\to 2\text{N}{{\text{H}}_{4}}^{+}+\text{S}{{\text{O}}_{4}}^{2-}$$

$\text{2N}{{\text{H}}_{4}}^{+}$ produced by (3) is taken up by roots; 2H^+^ is exuded from the roots to balance charges and reacts with the product of (3) as follows:

(4) $$2{\text{H}}^{+}+\text{S}{{\text{O}}_{4}}^{2-}\to {\text{H}}_{2}\text{S}{\text{O}}_{4}$$

(5) $$2{\text{H}}_{2}\text{S}{\text{O}}_{4}+\text{C}{\text{a}}_{2}\text{Si}{\text{O}}_{4}\to {\text{H}}_{4}\text{Si}{\text{O}}_{4}+\text{CaS}{\text{O}}_{4}$$

Importantly, the decreased adsorption of silicic acid onto clay particles at low pH (see above) would allow more of the solubilized Si to remain in the soil solution and be available for plant uptake.

To investigate the influence of N-form on soil pH and Si uptake, we conducted two trials using potted sugarcane plants. Both trials tested the primary hypothesis that Si uptake and cane yield following fertilization with Ca_2_SiO_4_ would be differentially affected by simultaneous treatment of plants with either $\text{N}{{\text{H}}_{4}}^{+}$ (rhizosphere acidification) or $\text{N}{{\text{O}}_{3}}^{-}$ (rhizosphere alkalinization) as N-form treatments. In addition, the second trial tested the hypothesis that Si uptake and cane yield responses to Ca_2_SiO_4_ application under these N-form treatments would differ between an N-efficient, more acid-tolerant cultivar and an N-inefficient, less acid-tolerant cultivar. Specifically, we predicted that an N-efficient, more acid-tolerant cultivar may yield better than its counter-part due to its lower demand for N (Meyer *et al.* 2007) and greater tolerance of the more acidic soil environment produced by $\text{N}{{\text{H}}_{4}}^{+}$ fertilization, while benefitting from the predicted greater availability of Si at lower soil pH. Under the weathered and acidic soil conditions of the rainfed areas of the South African sugar industry, such cultivar characteristics are advantageous and may ensure that greater benefits are derived from soil Si amendments.

To our knowledge, the present work is one of only three published studies (the others being that of Oliveira *et al.* 2007 on rice, and Leusch and Buchenauer 1989 on wheat (*Triticum* spp.)) that have addressed the effect of fertilizer N-form on Si uptake in any plant and the first to examine whether this effect may vary significantly between cultivars.

## Methods

### Trial establishment

The two trials were conducted in a shadehouse with clear polycarbonate roofing and walls of 40 % green shade cloth, over two successive seasons (December 2009 and 2010). Pot trials were preferred to field trials as they afford superior control over extraneous conditions (and especially variability in soil composition) when basic principles, such as those studied here, are under investigation.

Sugarcane transplants were produced from single budded setts, cut from mature stalks in field-grown plots of commercial sugarcane of the same age and from the same field. For both trials, 1-month-old transplants were planted into 3.0 L plant pots (one seedling per pot) placed on individual drip trays. Each pot contained 3.5 kg (dry weight) of soil sieved through a 1.0-mm mesh. The required amount of soil was collected from the top 15-cm soil layer at the same locality (Gratton Farm, Eshowe, South Africa, 31°28′29″E; 28°51′21″S; 473 m absl) and from the same section of fallow field during November of each year. The soil type was a Glenrosa form, Inceptisol (Soil Survey Staff 2003), which in KwaZulu-Natal (South Africa) typically consists of grey loamy sands, moderately to strongly acid, with a general paucity of plant foods (Beater 1970). The soil was chosen as it is typical of the weathered, comparatively high acid saturation (i.e. high soluble Al) and low-Si soils of the rainfed regions of the South African sugar industry, as described earlier. The characteristics of the soil, based on samples taken from the field in September 2009, are given in Table 1. Liming is recommended for soils in the industry with an acid saturation index >20 %, except for cv. N12 (used in this study; see below), where a 40 % threshold is applied (Schroeder *et al.* 1995).

**Table 1.** Characteristics of soil from Gratton Farm (Eshowe, South Africa) used in Trials 1 and 2 (mean values from five field samples, standard errors in parentheses). ^a^Aluminium saturation index; ^b^organic matter.

P	K	Ca	Mg	Si	pH (water)	ASI (%)^a^	OM (%)^b^	Clay (%)	Sand (%)	Silt (%)
59.2 (5.7)	69.0 (2.3)	109.8 (12.8)	36.7 (3.2)	8.4 (0.7)	5.0 (0.04)	26.4 (3.3)	1.3 (0.1)	8.8 (0.6)	83.4 (0.4)	7.4 (0.6)

Calcium silicate slag (Calmasil^®^, a stainless steel slag with 10.3 % Si; supplied by PDB Holdings, Pty Ltd, Middelburg, South Africa) was supplied to all pots as a Si fertilizer by thoroughly incorporating it into the soil on a pot-by-pot basis at 12 g pot^−1^ (=3429 mg Si kg^−1^), which was equivalent to a product rate of 7.4 tons ha^−1^ and a Si rate of 757 kg ha^−1^. Trials 1 and 2 were planted 3 and 6 days, respectively, after Ca_2_SiO_4_ incorporation, followed by application of N treatments (see below) in aqueous solution at 200 mL pot^−1^. Soil analyses (of five samples from the soil collected) by the South African Sugarcane Research Institute's (SASRI) Fertilizer Advisory Service (FAS) indicated that potassium (K) was required at 150 kg ha^−1^ as well as N at 120 kg ha^−1^. However, previous research (Fageria 2005) and our own experience have shown that fertilizer rates in pot trials need to be substantially increased above their field equivalents to ensure that leaf nutrient thresholds are attained. Therefore, K was applied to all pots in both trials as potassium chloride (KCl) at 300 kg K ha^−1^ (0.94 g KCl pot^−1^). Nitrogen rates were similarly increased above those recommended for the field (see below). No other nutrient supplements were required.

Plants were watered daily to every third day with 100–500 mL at a time, depending on growth stage and ambient temperatures. Overflow of leachate from drip trays was avoided to prevent nutrient loss. Pots were spaced 1 m apart to allow easy access and to ensure plants had good light exposure.

### Treatments and design

In Trial 1, three N treatments were balanced to provide 300 kg N ha^−1^ to all pots as follows: T1: (NH_4_)_2_SO_4_ (2.38 g pot^−1^); T2: ammonium thiosulphate [(NH_4_)_2_S_2_O_3_] (2.67 g pot^−1^) + dicyandiamide (DCD; 15 mg pot^−1^); T3: calcium nitrate [Ca(NO_3_)_2_] (4.25 g pot^−1^). Thiosulphate and DCD were used for T2 as they are both nitrification inhibitors and prevented the bacterial conversion of $\text{N}{{\text{H}}_{4}}^{+}$ to $\text{N}{{\text{O}}_{3}}^{-}$, while Treatment T1 was included to control for any potential effects of DCD on Si uptake. All pots were planted to the commercial cultivar N11 (Anon. 1986). Comparisons of T1 and T2 with T3 tested the primary hypothesis that Si uptake would be affected by different N-form treatments.

Based on the results of Trial 1, which showed no effects of DCD on Si uptake in T2 (see Results), DCD was excluded from Trial 2 and N rates were adjusted to 210 kg N ha^−1^ across all pots. The latter was done as many of the leaf N % values obtained in Trial 1 were >2.0 % (threshold = 1.8 %; Miles and Rhodes 2013). In Trial 2, two N treatments T1 and T2 were applied as 1.85 g pot^−1^ (NH_4_)_2_S_2_O_3_ and 2.97 g pot^−1^ Ca(NO_3_)_2_, respectively. Cultivar treatments included an N-efficient, more acid-tolerant cultivar (N12) (Anon. 2006*a*) and an N-inefficient, less acid-intolerant cultivar (N14) (Anon. 2006*b*). In this instance, comparisons of T1 with T2 tested the primary hypothesis (that N-form affects Si uptake) and also tested for a possible interaction between N-form and cultivar.

Treatments were arranged in a fully factorial randomized block design with 12 replications (36 pots) in Trial 1 and 10 replications (40 pots) in Trial 2.

### Data collection and analysis

#### Soil analysis

Trial 1 was harvested at 18 weeks and Trial 2 at 20 weeks. Trial duration was limited to this period to avoid root binding and water stress in the relatively small pots; use of larger pots was constrained by soil transport costs and logistics. Soil samples were taken individually from half the total number of pots (i.e. replicates) in each trial to produce six samples per treatment for Trial 1 and five samples per treatment for Trial 2. Samples were submitted to the SASRI FAS for pH and Si determination. In Trial 1, soil Si was extracted using 0.02 N sulphuric acid (H_2_SO_4_) (Kanamugire *et al.* 2006) only, while in Trial 2, 0.02 N H_2_SO_4_ and 0.01 M calcium chloride (CaCl_2_) were used. The latter extraction method is now under general international use for determining readily available Si in soil solution, with satisfactory correlations with plant uptake; stronger acid extractants often show much weaker relationships with plant uptake as they also solubilize Si that is more strongly adsorbed onto sesquioxides and not readily available (Berthelsen *et al.* 2001*b*; Berthelsen and Korndörfer 2005; Sauer *et al.* 2006). Silicon concentrations were determined photometrically using the ammonium molybdate method described by Fox *et al.* (1969). pH was measured in water.

#### Leaf, stalk and yield analysis

Leaf samples (third fully unfurled or ‘top visible dewlap’ leaf) were taken at trial harvest from the major tillers in each pot; leaf blades were stripped from the midrib and the blades dried, ground and submitted as separate samples per pot for Si and N analysis by the SASRI FAS. Thereafter, all tillers were removed at the base, stripped of all leaf material and the stalks chopped into pieces ∼1 cm long. The leaves from each pot were combined into bundles, while the chopped stalk pieces from each pot were combined into separate paper packets. All the material was dried to constant weight in an oven at 60 °C and the dry mass of leaf and stalk material determined individually for each pot. The dried stalk material was subsequently ground and sieved (1 mm mesh) for determination of Si content in Trial 1, and Si and N [using Kjeldahl method for N; (Sahrawat *et al.* 2002)] content in Trial 2, separately for each pot. Plant (leaf and stalk) Si content was determined photometrically using the dry ashing and ammonium molybdate method of Fox *et al.* (1969).

#### Statistical analysis

All data were tested for univariate normality (Anderson Darling or Shapiro–Wilk tests) and homogeneity of variance (Bartlett's test) prior to analysis of variance (ANOVA). Where conditions for parametric analysis were not met, log_10_ transformations were applied prior to ANOVA. Rather than back-transforming means, raw means (and their standard errors) for treatments were calculated for the purposes of presentation. Planned comparisons of means were performed using Fisher's protected least-significant difference (LSD) test. Linear regression analysis was performed using individual pot values across all treatments within each trial to examine relationships between soil pH, soil Si, leaf Si and stalk Si concentrations. GenStat 14th edn and SigmaPlot 12.5 were used for analyses.

## Results

### Soil analysis

**Figure 1.**
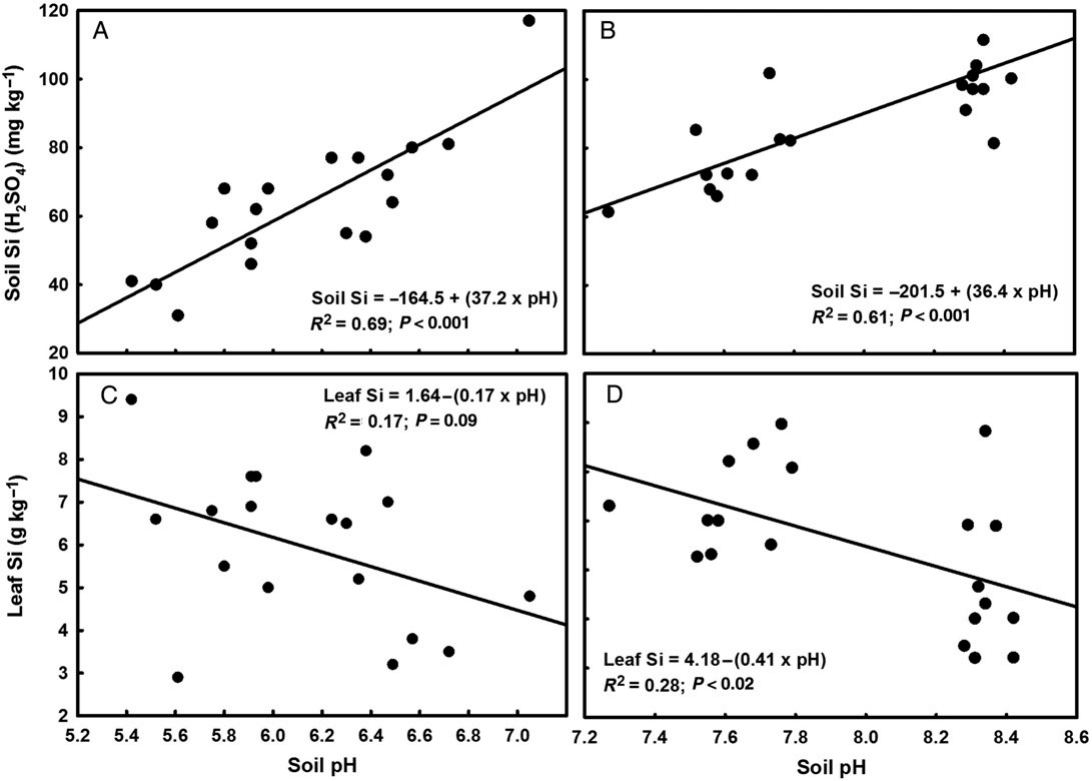
Regression curves and equations for 0.02 N H_2_SO_4_-extractable soil Si concentration in Trial 1 (A) and Trial 2 (B), and leaf Si concentration in Trial 1 (C) and Trial 2 (D), against soil pH at trial harvest (18 weeks for Trial 1 and 20 weeks for Trial 2). Data points are individual pot values. Note the different pH scales for Trials 1 and 2.

The $\text{N}{{\text{H}}_{4}}^{+}$ treatments lowered pH significantly compared with the $\text{N}{{\text{O}}_{3}}^{-}$ treatment in both trials (Table 2). In Trial 1, soil Si extracted with 0.02 N H_2_SO_4_ was significantly lower in Treatment T2 than in T1 and T3. While soil Si in T1 was also lower than that of T3, the two treatments did not differ significantly (Table 2). In Trial 2, Si extracted using H_2_SO_4_ was significantly less in Treatment T1 than in T2 (Table 2), while values between T1 and T2 did not differ significantly using 0.01M CaCl_2_ as an extractant (Table 2). It is notable that both soil Si and pH were appreciably higher in Trial 2 treatments compared with their equivalents in Trial 1 (Table 2; Fig. 1).

**Table 2.** Soil analysis for pH and 0.02 N H_2_SO_4_-extractable and 0.01 M CaCl_2_-extractable Si content in Trials 1 and 2 after harvest (18 weeks for Trial 1 and 20 weeks for Trial 2). ^a^CaCl_2_ extraction was not performed in Trial 1. ^b^‘*T*’ = N-form treatment. *N* = 6 (Trial 1) and 5 (Trial 2). Probability (*P*) values are from ANOVA. Means within the same column followed by the same letter are not significantly different (Fisher's protected LSD, *P* < 0.05).

Treatment/statistic	pH	Si (mg kg^−1^)^a^	
		H_2_SO_4_	CaCl_2_
Trial 1			
T1 [(NH_4_)_2_SO_4_]	6.2 ± 0.1ab	64.8 ± 4.3b	–
T2 [(NH_4_)_2_S_2_O_3_+ DCD]	5.7 ± 0.1a	51.5 ± 4.7a	–
T3 [Ca(NO_3_)_2_]	6.5 ± 0.2b	74.3 ± 4.0b	–
*P* value	0.02	0.02	
Trial 2			
T1 [(NH_4_)_2_S_2_O_3_]	7.6 ± 0.05	76.4 ± 3.7	20.2 ± 0.7
T2 [Ca(NO_3_)_2_]	8.3 ± 0.02	101.9 ± 4.6	22.5 ± 1.5
*P* value	<0.001	<0.001	0.2
Cultivar N12	7.9 ± 0.1	89.6 ± 7.4	22.4 ± 1.2
Cultivar N14	8.0 ± 0.1	88.7 ± 4.1	20.3 ± 1.1
*P* value	0.03	0.9	0.2
*P* value: *T*^b^× cultivar	0.08	0.09	0.5

**Figure 2.**
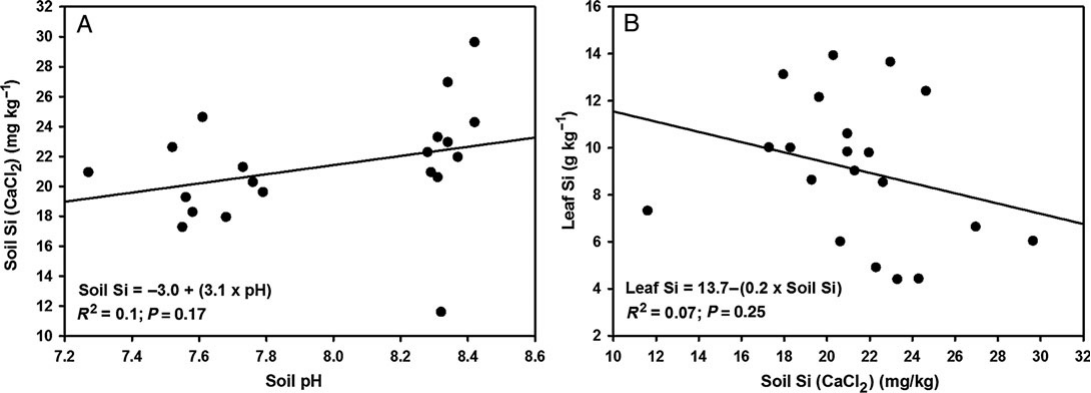
Regression curves and equations for 0.01 M CaCl_2_-extractable soil Si against soil pH (A) and leaf Si against 0.01 M CaCl_2_-extractable soil Si (B) in Trial 2 at harvest (20 weeks). Data points are individual pot values.

There was a significant positive relationship between soil pH_water_ and H_2_SO_4_-extractable soil Si in both trials (Fig. 1A and B), but no relationship between pH and CaCl_2_-extractable soil Si (Fig. 2A).

Cultivar did not affect soil Si content (both extractants), but pH was significantly higher for cv. N14 than for cv. N12 (Table 2). There were no significant N treatment × cultivar interactions for pH or soil Si.

### Leaf, stalk and yield analysis

**Figure 3.**
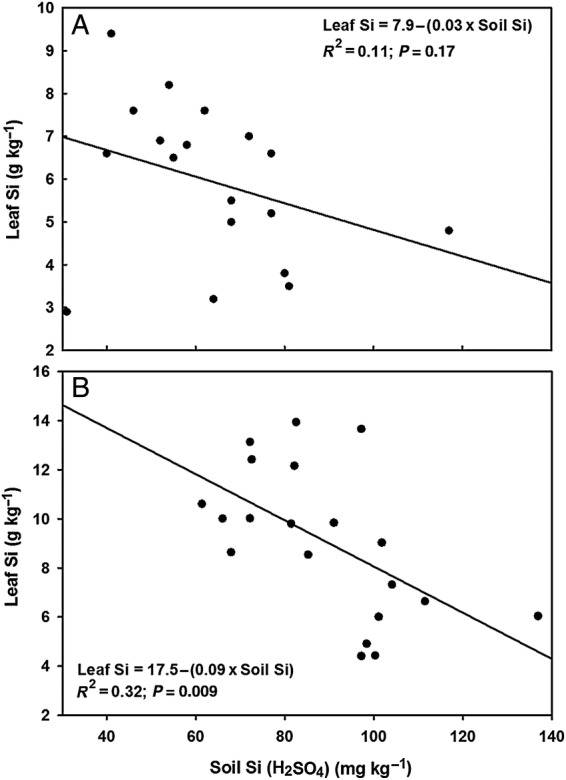
Regression curves and equations for leaf Si concentration in Trial 1 (A) and Trial 2 (B) against 0.02 N H_2_SO_4_-extractable soil Si concentration at trial harvest (18 weeks for Trial 1 and 20 weeks for Trial 2). Data points are individual pot values.

Leaf and stalk Si content increased significantly in the $\text{N}{{\text{H}}_{4}}^{+}$ treatments compared with the $\text{N}{{\text{O}}_{3}}^{-}$ treatments in both trials, with no effect from the inclusion of a nitrification inhibitor (Table 3). There was no effect of cultivar on leaf or stalk Si content (Table 3). A significant negative relationship occurred between leaf Si content and soil pH at harvest in Trial 2 (Fig. 1D), but not in Trial 1 (Fig. 1C) where the relationship between the two variables was weakly negative. There was also a significant negative relationship between leaf Si content and H_2_SO_4_-extractable soil Si at harvest in Trial 2 (Fig. 3B), but not in Trial 1 (Fig. 3A) where the relationship was weakly negative; there was no significant relationship between leaf Si content and CaCl_2_-extractable soil Si in Trial 2 (Fig. 2B).

**Table 3.** Leaf and stalk Si and N content in Trials 1 and 2 at harvest (18 weeks for Trial 1 and 20 weeks for Trial 2). Values are means ± standard error. ^a^‘*T*’ = N-form treatment. *N* = 12 (Trial 1) and 10 (Trial 2). Probability (*P*) values are from ANOVA; NS, not significant. Means within the same column followed by the same letter are not significantly different (Fisher's protected LSD, *P* < 0.05).

Treatment/statistic	Leaf		Stalk	
	Si (g kg^−1^)	N (g kg^−1^)	Si (g kg^−1^)	N (g kg^−1^)
Trial 1				
T1 [(NH_4_)_2_SO_4_]	7.1 ± 0.4a	18.6 ± 0.7	11.1 ± 0.6a	–
T2 [(NH_4_)_2_S_2_O_3_+ DCD]	7.3 ± 0.7a	19.6 ± 0.4	11.6 ± 0.9a	–
T3 [Ca(NO_3_)_2_]	5.4 ± 0.7b	19.1 ± 0.5	8.2 ± 0.7b	–
*P* value	<0.05	0.4	<0.001	–
Trial 2				
T1 [(NH_4_)_2_S_2_O_3_]	10.8 ± 0.4	8.5 ± 0.6	6.4 ± 0.3	2.6 ± 0.4
T2 [Ca(NO_3_)_2_]	7.0 ± 0.5	11.2 ± 0.7	5.0 ± 0.3	5.3 ± 0.5
*P* value	<0.001	<0.01	0.002	<0.001
Cultivar N12	8.4 ± 0.5	9.7 ± 0.7	5.5 ± 0.3	4.7 ± 0.5
Cultivar N14	9.3 ± 0.7	9.9 ± 0.7	5.9 ± 0.3	3.3 ± 0.4
*P* value	0.2	0.8	0.3	<0.02
*P* value: *T*^a^ × cultivar	0.6	0.1	0.9	0.9

**Figure 4.**
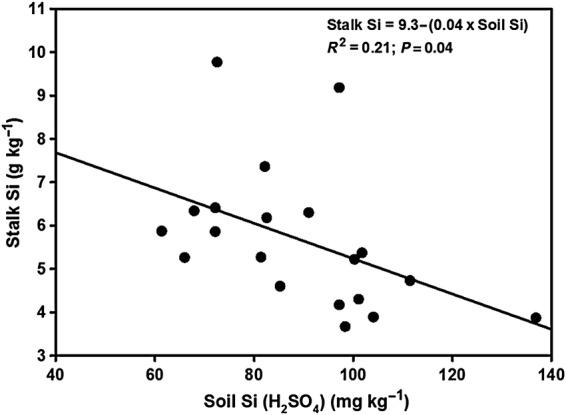
Regression curve and equation for stalk Si concentration against 0.02 N H_2_SO_4_-extractable soil Si concentration in Trial 2 at harvest (20 weeks). Data points are individual pot values.

In Trial 2, stalk Si content was significantly negatively related to H_2_SO_4_-extractable soil Si (Fig. 4). Leaf and stalk Si content were significantly positively correlated (*r* = 0.78, *P* < 0.001, *N* = 20, Pearson's correlation).

We found no effect of the N treatments on leaf N content in Trial 1 (Table 3). However, leaf N was significantly greater in T2 than in T1 in Trial 2 (Table 3); furthermore, stalk N content was significantly increased in T2 in this trial (Table 3).

Although cultivar did not affect leaf N content in Trial 2, stalk N in this trial was significantly greater in cv. N12 than in cv. N14 (Table 3). There were no significant interactions between N treatment and cultivar for leaf Si and leaf N, or stalk Si and stalk N in Trial 2 (Table 3).

Neither the N treatments nor cultivar treatments affected dry leaf or stalk mass at harvest (Table 4). There were no significant interactions between N treatment and cultivar for both yield parameters (Table 4).

**Table 4.** Dry leaf and stalk mass for Trials 1 and 2 at harvest (18 weeks for Trial 1 and 20 weeks for Trial 2). Values are means ± standard error. ^a^‘*T*’ = N-form treatment. *N* = 12 (Trial 1) and 10 (Trial 2). Probability (*P*) values are from ANOVA.

Treatment/statistic	Leaf (g)	Stalk (g)
Trial 1		
T1 [(NH_4_)_2_SO_4_]	4.6 ± 0.2	17.3 ± 0.7
T2 [(NH_4_)_2_S_2_O_3_+ DCD]	4.7 ± 0.3	17.5 ± 1.1
T3 [Ca(NO_3_)_2_]	3.9 ± 0.2	18.1 ± 0.6
*P* value	0.1	0.2
Trial 2		
T1 [(NH_4_)_2_S_2_O_3_]	31.0 ± 0.9	21.8 ± 0.9
T2 [Ca(NO_3_)_2_]	33.2 ± 0.9	21.2 ± 1.3
*P* value	0.1	0.7
Cultivar N12	30.9 ± 1.0	22.1 ± 1.2
Cultivar N14	33.3 ± 0.8	20.9 ± 0.9
*P* value	0.06	0.4
*P* value: *T*^a^ × cultivar	0.7	0.5

## Discussion

Our results support the hypothesis that reduced soil pH, resulting either from bulk acidification of soil due to nitrification of $\text{N}{{\text{H}}_{4}}^{+}$ supplied as (NH_4_)_2_SO_4_ or (NH_4_)_2_S_2_O_3_ or from H^+^ ions exuded from the root to balance charges after $\text{N}{{\text{H}}_{4}}^{+}$ uptake, increased the solubilization of Si from Ca_2_SiO_4_. As our treatments included a nitrification inhibitor (DCD or (NH_4_)_2_S_2_O_3_), it is likely that the balancing of cation charges through H^+^ exudation into the rhizosphere (Equations (3)–(5)) was the dominant mechanism for reducing rhizosphere pH under our experimental conditions. Under field conditions, both mechanisms could play a role, with their relative contributions depending on soil characteristics (e.g. pH buffering capacity) and cultivar (Haynes 1990; Hinsinger *et al.* 2003). This is the first published study to demonstrate that application of N in the form of $\text{N}{{\text{H}}_{4}}^{+}$ and an associated reduction in rhizosphere pH increased plant uptake of Si from an applied source.

Our results indicate that sugarcane cultivars can influence soil pH and that cv. N12 (the most acid- and Al-tolerant of South African sugarcane cultivars; Schroeder *et al.* 1995) may have a greater capacity to acidify the rhizosphere via H^+^ exudation than cv. N14. Increased dissociation of Ca_2_SiO_4_ under more acidic soil conditions, as in Reaction B, would increase the availability of silicic acid for plant uptake, especially when this occurs in the immediate vicinity of the roots, as in Equations (3)–(5). Our study found no support for the alternative hypothesis that rhizosphere alkalinization, as a result of fertilization with $\text{N}{{\text{O}}_{3}}^{-},$ increased Si uptake into leaves or stalk. Our results concur with those of Leusch and Buchenauer (1989), who reported significantly increased Si uptake by wheat from applied silicate slag when the plants were fertilized with N as $\text{N}{{\text{H}}_{4}}^{+}$ rather than $\text{N}{{\text{O}}_{3}}^{-}$.

There was also no evidence for a differential effect (i.e. no significant interaction) of N-form on Si uptake between cultivars, and cultivars showed no significant differences in leaf or stalk Si content. Hence, despite the lower soil pH associated with cv. N12, we cannot conclude that this N-efficient, more acid-tolerant cultivar (Schroeder *et al.* 1995; Schumann *et al.* 1998) is likely to take up more Si under low soil pH conditions than an N-inefficient, less acid-tolerant cultivar (cv. N14; Schumann *et al.* 1998). However, in line with its greater N-efficiency, cv. N12 exhibited a higher stalk N content, although this did not manifest itself as increased yield in this cultivar compared with cv. N14.

As the $\text{N}{{\text{O}}_{3}}^{-}$ treatment significantly increased leaf and stalk N content in Trial 2, it might be argued that plant Si content was reduced under the $\text{N}{{\text{O}}_{3}}^{-}$ treatment due to a dilution effect resulting from enhanced growth under greater N uptake (e.g. Fallah 2012; Artigiani *et al.* 2014; Tsujimoto *et al.* 2014). However, no significant differences in plant dry mass were recorded between N treatments, indicating that the higher Si levels under the $\text{N}{{\text{H}}_{4}}^{+}$ treatment were not a byproduct of a reduced dilution effect in this treatment.

The lack of a yield difference between N-form treatments, notwithstanding the difference in leaf and stalk tissue Si content, may be due to the absence of imposed stress. There is increasing evidence that Si amendment—due to its action in plant stress alleviation—has significant effects on yield only in the presence of biotic and/or abiotic stress (reviews by Ma 2004; Epstein 2009; Wu *et al.* 2013; Haynes 2014; Zhu and Gong 2014). Thus, in mature field-grown sugarcane subject to crop stress (e.g. water deficit, pests or disease), the economic benefits of Si amendment and enhanced uptake are more likely to be expressed (Meyer and Keeping 2005).

Our findings agree with those of Oliveira *et al.* (2007) for upland rice to the extent that these authors found that application of N at a higher ratio of NH_4_^+^ to $\text{N}{{\text{O}}_{3}}^{-}$ progressively reduced soil pH and was associated with lower 0.5 M CaCl_2_-extractable soil Si. However, our results contrast with theirs for plant uptake of Si in relation to soil pH (see Fig. 1), which in Oliveira *et al.*'s (2007) study increased significantly with increasing rhizosphere pH. Notwithstanding this relationship, their Fig. 6 indicates a positive relationship between the $\text{N}{{\text{H}}_{4}}^{+}:\text{N}{{\text{O}}_{3}}^{-}$ ratio and the shoot Si content, which is contradictory to the positive relationship they found between Si uptake and soil pH (their Fig. 5) (since the pH decreased with increasing NH_4_^+^ provision).

Oliveira *et al.* (2007), Korndörfer *et al.* (2005) and de Camargo *et al.* (2007) argued that the concentration of available soil Si (whether native Si or added as an amendment) decreases with increasing soil acidity (Korndörfer *et al.* 2005). However, numerous studies have found that the solubility of silicic acid in the soil solution decreases with increasing pH up to a value of 9.8 (the pK_1_ for dissociation of silicic acid, H_4_SiO_4_, to the silicate anion, ${\text{H}}_{3}\text{Si}{{\text{O}}_{4}}^{-}$ (Haynes 2014)), due to the preferential adsorption of ${\text{H}}_{3}\text{Si}{{\text{O}}_{4}}^{-}$ onto iron and Al sesquioxides (Beckwith and Reeve 1963, 1964; Jones and Handreck 1963; McKeague and Cline 1963*a*, *b*; Hingston and Raupach 1967; Kato and Owa 1996; Tavakkoli *et al.* 2011; Makabe-Sasaki *et al.* 2013). In mature soils, sesquioxides form the most important general source of rapidly acid-soluble Si (Beckwith and Reeve 1964); consequently, highly weathered soils, such as that used in the present study, can rapidly remove ‘added’ Si from the soil solution through adsorption reactions, making it unavailable for plant uptake (Jones and Handreck 1965; Tavakkoli *et al.* 2011).

Notwithstanding the greater Si uptake in the NH_4_^+^ treatments, significantly less soil Si was extracted using 0.02 N H_2_SO_4_ from the $\text{N}{{\text{H}}_{4}}^{+}$ treatments than from the $\text{N}{{\text{O}}_{3}}^{-}$ treatments (especially in Trial 2; Table 2), and there was a significant positive association between acid-extractable Si and soil pH (Fig. 1). A likely explanation for this paradox is that under higher soil pH conditions of the $\text{N}{{\text{O}}_{3}}^{-}$ treatment, greater amounts of applied Si were present as the silicate anion and adsorbed on soil surfaces (i.e. removed from soil solution) than under the $\text{N}{{\text{H}}_{4}}^{+}$ treatment, making this fraction unavailable for plant uptake. However, this adsorbed fraction may be detectable at trial termination as significantly higher levels of Si in the $\text{N}{{\text{O}}_{3}}^{-}$ treatment when released into the solution by the relatively strong 0.02 N H_2_SO_4_ extraction. Soil Si values under the $\text{N}{{\text{H}}_{4}}^{+}$ treatment were likely further reduced by greater plant uptake compared with the $\text{N}{{\text{O}}_{3}}^{-}$ treatment, leaving less for extraction from the soil at trial termination.

Both processes may explain the significant negative association between acid-extractable soil Si at harvest and leaf Si content (Fig. 3B). The acid extraction apparently gave an unrealistically high measure of the plant-available Si in the $\text{N}{{\text{O}}_{3}}^{-}$ treatment during plant growth, a property of stronger acid extractants that has been well-documented (Berthelsen and Korndörfer 2005; Sauer *et al.* 2006; Haynes 2014). Extraction with 0.01 M CaCl_2_, on the other hand, yielded no significant difference in soil Si between $\text{N}{{\text{H}}_{4}}^{+}$ and $\text{N}{{\text{O}}_{3}}^{-}$ treatments, and no significant associations between soil Si and pH (Fig. 2A) or soil Si and leaf Si (Fig. 2B). This result is consistent with the above explanation, as 0.01 M CaCl_2_, being a dilute, neutral salt solution, would have extracted mostly soluble Si and less of the Si adsorbed to soil surfaces. The weakly negative association between CaCl_2_-extracted soil Si and leaf Si (Fig. 2B), rather than an expected positive association based on previous studies (e.g. Berthelsen *et al.* 2001*b*; de Camargo *et al.* 2007; Haynes *et al.* 2013; Miles *et al.* 2014), nonetheless indicates that this method solubilized more Si in the $\text{N}{{\text{O}}_{3}}^{-}$ treatment than was readily available for plant uptake.

While several other studies have reported significant positive correlations between soluble soil Si and pH in slag-amended soils (Korndörfer *et al.* 2005; de Camargo *et al.* 2007; Oliveira *et al.* 2007; Haynes *et al.* 2013), Haynes *et al.* (2013) and Haynes (2014) made the critical point that this relationship is probably coincidental rather than causative, as the continued dissolution of the slag material would inevitably raise soil pH while supplying extractable Si. Hence, an increase in soil pH *per se* may not enhance native or applied Si solubility, or significantly improve its uptake. For example, de Camargo *et al.* (2007) found that liming an acid, low-Si soil, had no effect on CaCl_2_-extractable soil Si and produced only a slight increase in plant Si compared with wollastonite and Ca-Mg silicate. In concurrence with our results, Khalid *et al.* (1978) and Tavakkoli *et al.* (2011), working in a sugarcane–corn–kikuyu grass rotation experiment and in rice, respectively, found that plant uptake of applied Si decreased with increasing soil pH, even though soil Si extracted with either phosphate solution (Khalid *et al.* 1978) or 0.01 M calcium chloride and 0.5 M ammonium acetate (Tavakkoli *et al.* 2011) increased with increasing soil pH. When these authors used water as an extractant, soluble Si decreased with increasing pH, which they ascribed to adsorption on soil surfaces (Khalid *et al.* 1978; Tavakkoli *et al.* 2011). However, the reaction of silicic acid with Al—the solubility of which is greatly increased at low pH values (<5.5)—to form insoluble hydroxyaluminosilicates is probably of particular importance in reducing Si solubility in acid soils (see Keeping *et al.* 2013; Haynes 2014, and references cited therein).

As both of our trials were planted only a few days after application of the Calmasil slag, it is likely that the slag was reacting (dissociating) during the entire period of the trials. The longer duration of Trial 2 (20 weeks) might therefore explain the much higher final pH and higher extractable Si in this trial compared with Trial 1 (18 weeks), and why $\text{N-N}{{\text{H}}_{4}}^{+}$ promoted its uptake to a greater degree in Trial 2 (Table 3, Fig. 2). Kato and Owa (1996, 1997) found that the increase in pH and Ca concentration of paddy soils following the addition of strongly alkaline Si-rich slags could suppress further dissolution of the slags as well as the soil Si concentration, the latter due to increased adsorption of Si onto the solid phase at higher pH. However, neutralization of soil pH (due to plant root and microbial respiration) increased Si dissolution from both the slags and the soil solid phase. Addition of N as $\text{N}{{\text{H}}_{4}}^{+}$ in the present study (as well as acid extraction for soil Si determination) likely achieved a similar result, suggesting that cultural practices, including addition of organic matter and associated increased microbial respiration, can be used to promote the dissolution of slags and improve crop Si uptake (Kato and Owa 1997; Ma and Takahashi 2002). Furthermore, a gradual increase in soil acidity due to nitrification of $\text{N}{{\text{H}}_{4}}^{+}$ fertilizers (urea is the most widely used N fertilizer in the South African sugar industry) and organic N is likely to release adsorbed Si from applied sources and make it available for plant uptake.

In addition to the potential use of $\text{N}{{\text{H}}_{4}}^{+}$ fertilizer in solubilizing applied Si, several studies have found that sugarcane accumulates $\text{N}{{\text{H}}_{4}}^{+}$ more efficiently as an N-source compared with $\text{N}{{\text{O}}_{3}}^{-}$ (de Armas *et al.* 1992; Robinson *et al.* 2011; Hajari *et al.* 2014). There is also evidence that $\text{N}{{\text{H}}_{4}}^{+}$ can significantly improve the use of other nutrients, especially P, by reducing rhizosphere pH and thereby increasing the solubility of P compounds, and also stimulating root growth (Jing *et al.* 2010, 2012). Together with the environmental and cost disadvantages of $\text{N}{{\text{O}}_{3}}^{-}$ arising from its high mobility and loss from the soil, an argument can be made for reducing $\text{N}{{\text{O}}_{3}}^{-}$ content in soils in favour of $\text{N}{{\text{H}}_{4}}^{+}$ and organic N-forms (Robinson *et al.* 2011), which are likely also to improve Si uptake.

An important distinction needs to be drawn between the enhancement of plant-available Si in association with raised soil pH following slag amendment of low-Si soils, and the abundance of native Si in higher pH, less-weathered soils, such as those that predominate in the northern irrigated regions of the South African sugar industry, where leaf Si levels are satisfactory (Van der Laan and Miles 2010). Miles *et al.* (2014) emphasize that this latter relationship between abundant available Si and higher pH is due to the occurrence of clays dominated by Si-rich minerals such as feldspars, vermiculites and smectites, which provide relatively high levels of soluble Si. In lower pH soils, such as those in the rainfed production areas, weathering processes have resulted in clay fractions dominated by low-Si minerals such as kaolinites and sesquioxides, while the high levels of exchangeable Al in such soils are also strongly associated with reduced acid- and CaCl_2_-extractable Si (Miles *et al.* 2014).

While weathering processes play a fundamental role in the development of desilicated soils, there is recent recognition of the effect of agriculture on terrestrial Si cycling, in particular with regard to biogenic silica (BSi; stored in biomass and soils as amorphous Si or plant phytoliths (SiO_2_·*n*H_2_O)). Struyf *et al.* (2010) and Vandevenne *et al.* (2012) showed that conversion of natural ecosystems to agriculture over centuries has disrupted cycling of BSi and created a new loop in the global Si cycle, wherein Si is exported from landscapes during agricultural harvest instead of being replenished through litterfall. Removal of Si through long-term cropping will ultimately deplete soil reserves of BSi, impacting both crop production and fluxes of Si from terrestrial environments into freshwater and oceanic systems (Struyf *et al.* 2010; Keller *et al.* 2012; Vandevenne *et al.* 2012). As SiO_2_ · *n*H_2_O solubility increases markedly with increasing pH, Haynes and Zhou (2014) argued that in the short term an increase in soil pH may increase Si availability in agricultural soils, but in the longer term it will reduce the available BSi-derived Si. In acidic soils where pH correction is necessary (e.g. through lime or slag application), replenishment of BSi through retention of crop residues may therefore be as important in preserving supplies of plant-available Si as the provision of Si in the form of silicate slags.

## Conclusions

There still appears to be some controversy surrounding the effects of soil pH on Si solubility, whether from native or applied Si. Our finding that Si uptake was significantly enhanced under lower pH caused by fertilization with $\text{N}{{\text{H}}_{4}}^{+},$ notwithstanding greater amounts of acid-extractable Si under higher pH produced by $\text{N}{{\text{O}}_{3}}^{-}$ fertilization, is worthy of further study at the soil chemistry level. We argue that the most likely explanation for our results is the reduction of rhizosphere pH from H^+^ extrusion to balance charges in response to $\text{N}{{\text{H}}_{4}}^{+}$ uptake, followed by greater solubilization (dissociation) of Ca_2_SiO_4_ in the more acidic root environment and production of plant-available silicic acid. In line with basic studies in the 1960s (Beckwith and Reeve 1963, 1964; Jones and Handreck 1963; McKeague and Cline 1963*c*), lower soil pH would also have reduced adsorption of Si to clay particles, especially Fe and Al sesquioxides, further increasing Si in soil solution. Results reported by other authors, such as Oliveira *et al.* (2007), which imply a direct positive effect of pH on Si solubility and uptake, may have misinterpreted the inevitable effect that addition of basic Si-rich slags has on soil pH while still raising soil Si concentration and therefore plant uptake (Haynes *et al.* 2013; Haynes 2014). Abundant native Si in the clays of higher-pH, less-weathered soils may also have contributed to the tenet that Si is more soluble in higher pH soils.

From an applied viewpoint, the use of ammoniacal N fertilizers to improve dissolution of applied slag and release readily available Si is worthy of field study. In addition to the provision of silicate materials to weathered Si-depleted soils, we advocate the preservation of the biogenic pool of Si as far as possible through the retention of crop residues and incorporation of organic matter.

## Sources of Funding

All funding for this study was provided by the South African Sugarcane Research Institute.

## Contributions by the Authors

The original concept for the study arose through discussion between R.S.R. and M.G.K. Calculations for treatments were performed by R.S.R., while the study itself was managed by M.G.K. Trial design and statistical analysis were performed by C.S. Debate over the findings and their interpretation were conducted between M.G.K., R.S.R. and N.M. The manuscript was written and revised by M.G.K. with criticisms and input from all other authors.

## Conflicts of Interest Statement

None declared.

## References

[PLU080C1] Alvarez J, Datnoff LE, Datnoff LE, Snyder GH, Korndörfer GH (2001). The economics of silicon for integrated management and sustainable production of rice and sugarcane. Silicon in agriculture. Studies in plant science.

[PLU080C2] Anon. (1986). *Sugarcane varieties grown in South Africa, bulletin no. 4* (*revised*.

[PLU080C3] Anon. (2006a). Information sheet 13.3 variety N12.

[PLU080C4] Anon. (2006b). Information sheet 13.4 variety N14.

[PLU080C6] Artigiani ACCA, Crusciol CAC, Nascente AS, Arf O, de Alvarez R (2014). Silicon in row and nitrogen in topdressing fertilization on rice under dry land and sprinkler irrigation conditions. Bioscience Journal.

[PLU080C7] Beater BE (1970). Soil series of the natal sugar belt.

[PLU080C8] Beckwith RS, Reeve R (1963). Studies on soluble silica in soils. I. The sorption of silicic acid by soils and minerals. Australian Journal of Soil Research.

[PLU080C9] Beckwith RS, Reeve R (1964). Studies on soluble silica in soils. II. The release of monosilicic acid from soils. Australian Journal of Soil Research.

[PLU080C10] Berthelsen S, Korndörfer GH (2005). Methods for Si analysis in plant, soil and fertilizers. *Proceedings of the Third Silicon in Agriculture Conference*.

[PLU080C11] Berthelsen S, Hurney A, Noble AD, Rudd A, Garside AL, Henderson A (2001a). An assessment of current silicon status of sugar cane production soils from Tully to Mossman. Proceedings of the Australian Society of Sugar Cane Technologists.

[PLU080C12] Berthelsen S, Noble AD, Garside AL, Datnoff LE, Snyder GH, Korndörfer GH (2001b). Silicon research down under: past, present, and future. Silicon in agriculture. Studies in plant science.

[PLU080C5] de Armas R, Valadier MH, Champigny ML, Lamaze T (1992). Influence of ammonium and nitrate on the growth and photosynthesis of sugarcane. Journal of Plant Physiology.

[PLU080C13] de Camargo MS, Pereira HS, Korndörfer GH, Queiroz AA, Reis CB (2007). Soil reaction and absorption of silicon by rice. Scientia Agricola.

[PLU080C14] Du Preez P (1970). The effect of silica on cane growth. Proceedings of the South African Sugar Technologists’ Association.

[PLU080C15] Epstein E, Datnoff LE, Snyder GH, Korndörfer GH (2001). Silicon in plants: facts vs. concepts. Silicon in agriculture. Studies in plant science.

[PLU080C16] Epstein E (2009). Silicon: its manifold roles in plants. Annals of Applied Biology.

[PLU080C17] Fageria NK (2005). Soil fertility and plant nutrition research under controlled conditions: basic principles and methodology. Journal of Plant Nutrition.

[PLU080C18] Fallah A (2012). Study of silicon and nitrogen effects on some physiological characters of rice. International Journal of Agriculture and Crop Sciences.

[PLU080C19] Fox RL, Silva JA, Plucknett DL, Teranishi DY (1969). Soluble and total silicon in sugar cane. Plant and Soil.

[PLU080C20] Guntzer F, Keller C, Meunier JD (2012). Benefits of plant silicon for crops: a review. Agronomy for Sustainable Development.

[PLU080C21] Hajari E, Snyman SJ, Watt MP (2014). Inorganic nitrogen uptake kinetics of sugarcane (*Saccharum* spp.) varieties under *in vitro* conditions with varying N supply. Plant Cell, Tissue and Organ Culture.

[PLU080C22] Haynes RJ (1990). Active ion uptake and maintenance of cation–anion balance: a critical examination of their role in regulating rhizosphere pH. Plant and Soil.

[PLU080C23] Haynes RJ (2014). A contemporary overview of silicon availability in agricultural soils. Journal of Plant Nutrition and Soil Science.

[PLU080C24] Haynes RJ, Zhou Y-F, Greger M (2014). Unravelling the enigma of the effect of soil pH on Si availability. Proceedings of the Sixth International Conference on Silicon in Agriculture.

[PLU080C25] Haynes RJ, Belyaeva ON, Kingston G (2013). Evaluation of industrial wastes as sources of fertilizer silicon using chemical extractions and plant uptake. Journal of Plant Nutrition and Soil Science.

[PLU080C26] Hingston FJ, Raupach M (1967). The reaction between monosilicic acid and aluminium hydroxide. I. Kinetics of adsorption of silicic acid by aluminium hydroxide. Australian Journal of Soil Research.

[PLU080C27] Hinsinger P, Plassard C, Tang C, Jaillard B (2003). Origins of root-mediated pH changes in the rhizosphere and their responses to environmental constraints: a review. Plant and Soil.

[PLU080C28] Jing J, Rui Y, Zhang F, Rengel Z, Shen J (2010). Localized application of phosphorus and ammonium improves growth of maize seedlings by stimulating root proliferation and rhizosphere acidification. Field Crops Research.

[PLU080C29] Jing J, Zhang F, Rengel Z, Shen J (2012). Localized fertilization with P plus N elicits an ammonium-dependent enhancement of maize root growth and nutrient uptake. Field Crops Research.

[PLU080C30] Jones LHP, Handreck KA (1963). Effects of iron and aluminium oxides on silica in solution in soils. Nature.

[PLU080C31] Jones LHP, Handreck KA (1965). Studies of silica in the oat plant III. Uptake of silica from soils by the plant. Plant and Soil.

[PLU080C32] Kanamugire A, Meyer JH, Haynes RJ, Naidoo G, Keeping MG (2006). An assessment of soil extraction methods for predicting the silicon requirement of sugarcane. Proceedings of the South African Sugar Technologists’ Association.

[PLU080C33] Kato N, Owa N (1996). The factors affecting Si concentration in the soil solution: effects of soil solution pH, Ca concentration, CO_2_ gas and slag application. Japanese Journal of Soil Science and Plant Nutrition.

[PLU080C34] Kato N, Owa N (1997). Dissolution of slag fertilizers in a paddy soil and Si uptake by rice plant. Soil Science and Plant Nutrition.

[PLU080C35] Keeping MG, Meyer JH, Sewpersad C (2013). Soil silicon amendments increase resistance of sugarcane to stalk borer *Eldana saccharina* Walker (Lepidoptera : Pyralidae) under field conditions. Plant and Soil.

[PLU080C36] Keller C, Guntzer F, Barboni D, Labreuche J, Meunier JD (2012). Impact of agriculture on the Si biogeochemical cycle: input from phytolith studies. Comptes Rendus Geoscience.

[PLU080C37] Khalid RA, Silva JA, Fox RL (1978). Residual effects of calcium silicate in tropical soils: I. Fate of applied silicon during five years cropping. Soil Science Society of America Journal.

[PLU080C38] Korndörfer GH, Nolla A, Ramos LA (2005). Available silicon in tropical soils and crop yield. *Proceedings of the Third Silicon in Agriculture Conference*.

[PLU080C78] Leusch HJ, Buchenauer H (1989). Einfluß von Bodenbehandlungen mit siliziumreichen Kalken und Natriumtrisilikat auf den Befall des Weizens mit *Erysiphe graminis* und *Septaria nodorum* in Abhängigkeit von der Form der N-Dünger. Zeitschrift für Pflanzenkrankheiten und Pflanzenschutz.

[PLU080C39] Liang YC, Sun WC, Zhu YG, Christie P (2007). Mechanisms of silicon-mediated alleviation of abiotic stresses in higher plants: a review. Environmental Pollution.

[PLU080C40] Ma JF (2004). Role of silicon in enhancing the resistance of plants to biotic and abiotic stresses. Soil Science and Plant Nutrition.

[PLU080C41] Ma JF, Takahashi E (2002). Soil, fertilizer and plant silicon research in Japan.

[PLU080C42] Ma JF, Miyake Y, Takahashi E, Datnoff LE, Snyder GH, Korndörfer GH (2001). Silicon as a beneficial element for crop plants. Silicon in Agriculture. Studies in plant science.

[PLU080C43] Makabe-Sasaki S, Kakuda K, Sasaki Y, Ando H (2013). Effect of slag silicate fertilizer on dissolved silicon in soil solution based on the chemical properties of Gleysols. Soil Science and Plant Nutrition.

[PLU080C44] Marafon AC, Endres L (2013). Silicon: fertilization and nutrition in higher plants. Revista de Ciências Agrárias/Amazonian Journal of Agricultural and Environmental Sciences.

[PLU080C45] Marschner H (1995). Mineral nutrition of higher plants.

[PLU080C46] McKeague JA, Cline MG (1963a). Silica in soil solutions I. The form and concentration of dissolved silica in aqueous extract of some soils. Canadian Journal of Soil Science.

[PLU080C47] McKeague JA, Cline MG (1963b). Silica in soil solutions II. The adsorption of monosilicic acid by soil and by other substances. Canadian Journal of Soil Science.

[PLU080C48] McKeague JA, Cline MG (1963c). Silica in soils. Advances in Agronomy.

[PLU080C49] Meyer JH, Keeping MG, Datnoff LE, Snyder GH, Korndörfer GH (2001). Past, present and future research of the role of silicon for sugarcane in southern Africa. Silicon in agriculture. Studies in plant science.

[PLU080C50] Meyer JH, Keeping MG (2005). An overview of the impact of silicon in overcoming biotic and abiotic stress in sugarcane. In: *Proceedings of the III Silicon in Agriculture Conference*.

[PLU080C51] Meyer JH, Harding R, Rampersad AL, Wood RA (1998). Monitoring long term soil fertility trends in the South African sugar industry using the FAS analytical database. Proceedings of the South African Sugar Technologists’ Association.

[PLU080C52] Meyer JH, Schumann AW, Wood RA, Nixon DJ, Berg M (2007). Recent advances to improve nitrogen use efficiency of sugarcane in the South African sugar industry. Proceedings of the International Society of Sugar Cane Technologists.

[PLU080C53] Miles N, Rhodes R (2013). Information sheet 7.17 guidelines for the interpretation of leaf analyses for sugarcane.

[PLU080C54] Miles N, Manson AD, Rhodes R, van Antwerpen R, Weigel A (2014). Extractable silicon in soils of the South African Sugar industry and relationships with crop uptake. Communications in Soil Science and Plant Analysis.

[PLU080C55] Oliveira LA, Korndörfer GH, Pereira AC (2007). Acumulação de silício em arroz em diferentes condições de pH da rizosfera. Revista Brasileira de Ciência do Solo.

[PLU080C56] Rhodes R, Miles N, Keeping MG (2013). Crop nutrition and soil textural effects on eldana damage in sugarcane. Proceedings of the South African Sugar Technologists’ Association.

[PLU080C57] Robinson N, Brackin R, Vinall K, Soper F, Holst J, Gamage H, Paungfoo-Lonhienne C, Rennenberg H, Lakshmanan P, Schmidt S (2011). Nitrate paradigm does not hold up for sugarcane. PLoS ONE.

[PLU080C58] Romero A, Munévar F, Cayón G (2011). Silicon and plant diseases. A review. Agronomía Colombiana.

[PLU080C59] Ross L, Nababsing P, Cheong YWY (1974). Residual effect of calcium silicate applied to sugarcane soils. Proceedings of the International Society of Sugar Cane Technologists.

[PLU080C60] Sahrawat KL, Kumar GR, Murthy KVS (2002). Sulfuric acid–selenium digestion for multi-element analysis in a single plant digest. Communications in Soil Science and Plant Analysis.

[PLU080C61] Sauer D, Saccone L, Conley DJ, Herrmann L, Sommer M (2006). Review of methodologies for extracting plant-available and amorphous Si from soils and aquatic sediments. Biogeochemistry.

[PLU080C62] Savant NK, Datnoff LE, Snyder GH (1997a). Depletion of plant-available silicon in soils: a possible cause of declining rice yields. Communications in Soil Science and Plant Analysis.

[PLU080C63] Savant NK, Snyder GH, Datnoff LE (1997b). Silicon management and sustainable rice production. Advances in Agronomy.

[PLU080C64] Savant NK, Korndörfer GH, Datnoff LE, Snyder GH (1999). Silicon nutrition and sugarcane production: a review. Journal of Plant Nutrition.

[PLU080C65] Schroeder BL, Turner P, Meyer JH (1995). Evaluation of a soil aluminium saturation index for use in the South African sugar belt. Proceedings of the South African Sugar Technologists’ Association.

[PLU080C66] Schumann AW, Meyer JH, Nair S (1998). Evidence for different nitrogen use efficiencies of selected sugarcane varieties. Proceedings of the South African Sugar Technologists’ Association.

[PLU080C67] Soil Survey Staff (2003). Keys to soil taxonomy.

[PLU080C68] Struyf E, Smis A, Van Damme S, Garnier J, Govers G, Van Wesemael B, Conley DJ, Batelaan O, Frot E, Clymans W, Vandevenne F, Lancelot C, Goos P, Meire P (2010). Historical land use change has lowered terrestrial silica mobilization. Nature Communications.

[PLU080C69] Tavakkoli E, Lyons G, English P, Guppy CN (2011). Silicon nutrition of rice is affected by soil pH, weathering and silicon fertilisation. Journal of Plant Nutrition and Soil Science.

[PLU080C70] Thomson CJ, Marschner H, Römheld V (1993). Effect of nitrogen fertilizer form on pH of the bulk soil and rhizosphere, and on the growth, phosphorus, and micronutrient uptake of bean. Journal of Plant Nutrition.

[PLU080C71] Tsujimoto Y, Muranaka S, Saito K, Asai H (2014). Limited Si-nutrient status of rice plants in relation to plant-available Si of soils, nitrogen fertilizer application, and rice-growing environments across Sub-Saharan Africa. Field Crops Research.

[PLU080C72] Van der Laan M, Miles N (2010). Nutrition of the South African sugar crop: current status and long-term trends. Proceedings of the South African Sugar Technologists’ Association.

[PLU080C73] Vandevenne F, Struyf E, Clymans W, Meire P (2012). Agricultural silica harvest: have humans created a new loop in the global silica cycle?. Frontiers in Ecology and the Environment.

[PLU080C74] Winslow MD (1992). Silicon, disease resistance, and yield of rice genotypes under upland cultural conditions. Crop Science.

[PLU080C75] Winslow MD, Okada K, Correa-Victoria F (1997). Silicon deficiency and the adaptation of tropical rice ecotypes. Plant and Soil.

[PLU080C77] Wu JW, Shi Y, Zhu YX, Wang YC, Gong HJ (2013). Mechanisms of enhanced heavy metal tolerance in plants by silicon: a review. Pedosphere.

[PLU080C76] Zhu YX, Gong HJ (2014). Beneficial effects of silicon on salt and drought tolerance in plants. Agronomy for Sustainable Development.

